# Decreased inflammatory gene expression accompanies the improvement of liver enzyme and lipid profile following aerobic training and vitamin D supplementation in T2DM patients

**DOI:** 10.1186/s12902-022-01152-x

**Published:** 2022-10-08

**Authors:** Rastegar Hoseini, Hiwa Ahmed Rahim, Jalal Khdhr Ahmed

**Affiliations:** 1 Department of Exercise Physiology, Faculty of Sport Sciences, Razi University, P.O.Box. 6714414971, Kermanshah, Iran; 2 Physical Education and Sport Sciences Department, University of Halabja, Halabja, Kurdistan Region 46018 Iraq

**Keywords:** Exercise, Inflammation, Diabetes, Vitamin D

## Abstract

**Background:**

Type 2 Diabetes Mellitus (T2DM) is one of the health issues causing untoward low-grade systemic inflammation. Aerobic Training (AT) and Vitamin D (Vit D) supplementation are among the approaches that improve lipid profile and liver enzymes in T2DM. However, the mechanisms responsible for these improvements are not fully elucidated.

**Objectives:**

This study aimed to evaluate the effects of AT and Vit D supplementation on lipid profile, liver enzymes, Interleukin-6 (IL-6), Interleukin-10 (IL-10), Cluster of differentiation 27 (CD27), Chemokine (C-X-C motif) Ligand 13 (CXCL13), Interferon-Gamma (IFN-γ) and Transforming Growth Factor-Beta 1 (TGF-β1) gene expressions in patients with T2DM.

**Methods:**

In this study, 40 male T2DM patients aged 35–50 years were randomly selected and assigned into four groups (*n* = 10 for each); AT+vitamin D supplementation (AT+Vit D), AT+placebo (AT), Vit D supplementation (Vit D), and control+placebo (C). The intervention consisted of 8 weeks of 20–40 minutes AT protocol at 60–75% HR_max_ 3 sessions/week and taking 50,000 IU of Vit D supplement once a week. Serum levels of lipid profile and liver enzymes and gene expression of IL-6, IL-10, CD27, CXCL13, IFN-γ, and TGF-β1 in Peripheral Blood Mononuclear Cells (PBMCs) were measured. One-way analysis of variance (ANOVA), Tukey’s post hoc, and paired sample t-test at *P*-values less than 0.05 were used to analyze the data using SPSS software.

**Results:**

AT+Vit D, AT, and Vit D significantly decreased TC, TG, LDL, AST, ALT, and GGT while increased HDL after 8 weeks in favor of AT+Vit D. Also, gene expressions of IL-6, IL-10, CD27, CXCL13, IFN-γ, and TGF-β1 were downregulated significantly in AT+Vit D, AT, and Vit D, while upregulated in C. Furthermore, compared to individual AT or Vit D, AT+Vit D significantly downregulated IL-6 (*P* = 0.013; *P* = 0.025), IL-10 (*P* = 0.012; *P* = 0.026), CD27 (*P* = 0.023; *P* = 0.041), CXCL13 (*P* = 0.014; *P* = 0.025), IFN-γ (*P* = 0.017; *P* = 0.026), and TGF-β1 (*P* = 0.001; *P* = 0.028).

**Conclusion:**

In comparison to individual AT or Vit D, AT+Vit D may enhance lipid profile, and liver enzymes and drive the balance to favor inhibition of inflammation by downregulating gene expression of inflammation-related factors. As a result, AT+Vit D may be considered appropriate therapy for managing T2DM.

## Introduction

Diabetes, particularly Type 2 Diabetes Mellitus (T2DM), is a health issue with many cardiovascular and neuromuscular diseases complications. Chronic hyperglycemia in diabetes gradually induces insulin resistance in many tissues, causing untoward low-grade systemic inflammation and creating insulin resistance-inflammation dual in diabetic patients. Several clinical trials have focused on non-pharmacological complementary approaches to prevent and control T2DM and its complication [1].

Accumulating evidence suggests that regular Aerobic Training (AT) helps facilitate skeletal muscle glucose uptake via insulin-dependent and noninsulin-dependent pathways, increasing GLUT4 abundance, boosting weight loss, and improving the liver enzyme and lipid profile [2, 3]. Thus, apart from these beneficial effects, AT may improve T2DM complications by targeting the possible mechanisms that drive the inflammatory pathways [4]. The mechanisms underlying AT-induced improvements are unknown. It is thought that AT, as a mechanical stimulus, may play a key role in modifying endothelial activity and the expression of a variety of proteins, particularly those involved in local inflammation (e.g., Interleukin-6 (IL-6), Interleukin-10 (IL-10), Cluster of Differentiation 27 (CD27), Chemokine (C-X-C motif) Ligand 13 (CXCL13). Studies investigating the effects of AT on inflammation in T2DM models are inconsistent [5, 6] and limited to serum level analyses [7, 8] or animal studies [9, 10].

Recent studies have focused on vitamin supplements (i.e., Vitamin D (Vit D)) in improving and controlling chronic disease. Additionally, studies reported the high prevalence of hypovitaminosis D in T2DM patients [11–13]. Beyond its captious role in the calcium-related insulin secretion [14], glucose transporter type 4 (GLUT-4) translocation [15], and glycemic status [12, 16], Vit D supplementation has also been found to play a critical role in the pathogenesis of the inflammatory disease via downregulating the production of the inflammatory cytokines [17].

Hoseini et al. [18] found that combined exercise and high dosages of Vit D supplementation lowered insulin, blood glucose, and homeostatic model evaluation for insulin resistance and increased peroxisome proliferator-activated receptor (PPAR) gene expression in ovariectomized rats. Also, Hoseini et al. [19] reported the combination of AT+ Vit D supplementation significantly reduced liver enzymes, anthropometric and glycemic indices, and improved lipid profile in older women with Vit D deficiency and NAFLD.

The literature supports the improvement of liver enzymes and lipid profiles after combined AT + Vit D supplementation and serum levels of inflammatory biomarkers after AT or Vit D supplementation in animal models of diabetes or other inflammation-related chronic diseases; however, few studies have investigated the effects of separate or combined AT and Vit D supplementation on gene expression of inflammatory biomarkers. Thus, we aimed at investigating the effects of AT and Vit D supplementation on IL-6, IL-10, CD27, CXCL13, IFN-, and TGF-1 gene expressions in patients with T2DM.

## Methodology

### Study design and participants

This is a randomized, single-blinded clinical trial with a placebo control conducted on 40 T2DM patients (over 3 years of diagnosed diabetes history) aged 35–50 years. This study was registered on the Iranian website for clinical trial registration IRCT20210811052151N1 on 01/09/2021, performed following the Declaration of Helsinki, and approved by the Research Ethics Committees of Kermanshah Razi University (IR.RAZI.REC.1400.044). All participants completed and signed written informed consent.

**Fig. 1:**
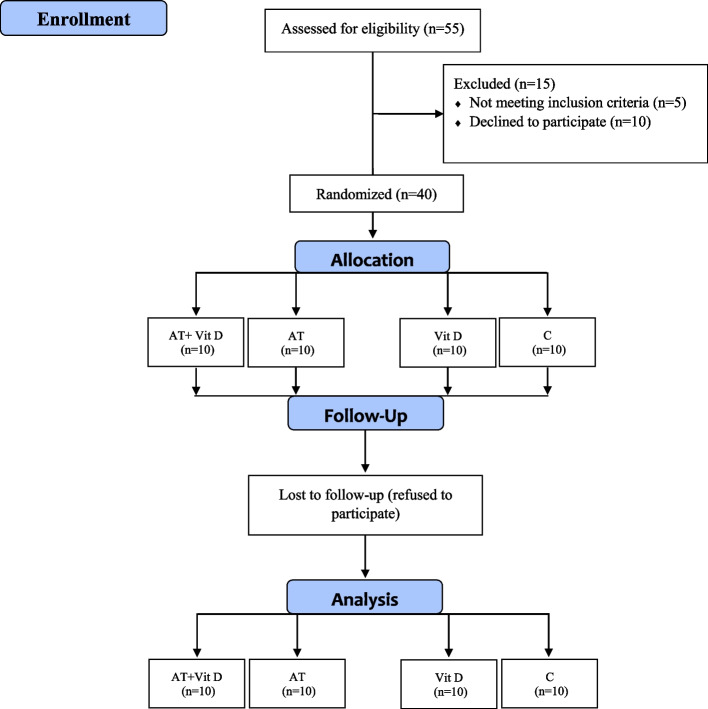
Flow chart of the study population

The main exclusion criteria included: more than 6 months of regular exercise schedule, history of orthopedic disorders, smoking, consumption of immunosuppressive drugs, muscle injuries, inability to perform the exercises, COVID-19 infection during the study period, taking anti-inflammatory and antioxidant supplements such as Vit D, E, and C, and omega-3 fatty acids within past 6 months. The sample size was evaluated at 36 subjects using G. POWER 3.1 software (alpha error of 0.05, power of 0.99, and effect size of 0.85). Due to the probability of refusal to continue the study, 40 male T2DM patients aged 35–50 years were randomly selected from the Diabetes Medical & Health Center. Then, they were randomly located in four groups: AT+ Vit D supplementation (AT+Vit D; *n* = 10), AT+placebo (AT; *n* = 10), Vit D supplementation (Vit D; *n* = 10), and control+placebo (C; *n* = 10) using the Random Number Generator method; all subjects had the same chance of being selected (Fig. 1).

#### Aerobic training

The AT and AT+Vit D groups participants were required to exercise 3 days per week for 8 weeks. The exercise training protocol started at 20 minutes of 60% HR_max_. It progressed to 40 minutes of 75% HR_max_ per session at the end of the eighth week, following the American Diabetes Association (ADA)'s guidelines [20]. Each session also included a 10-minute warmup and a 10-minute cooldown. A combination of the HR_max_ formula [HR_max_ = 220 − age] [21] and the 6–20 Rating of Perceived Exertion (RPE) scale were used in the outsets to control the intensity of the training sessions and ensure that the target heart rate (exercise intensity) was obtained and sustained during the study [22]. All participants learned the pulse palpation method to count their heart rates. Furthermore, the training sessions were conducted under the supervision of exercise physiologists (Table 1).

**Table 1:** The aerobic training

Variables	Week							
	1	2	3	4	5	6	7	8
**Intensity (HR**_**Max**_**)**	60–65%	60–65%	60–65%	65–70%	65–70%	65–70%	70–75%	70–75%
**Time (min)**	20	25	30	30	35	35	40	40
**Borg scale**	10	10	11	11	12	12	13	13

#### Diet and supplementation

In this study, Vit D and AT+Vit D groups consumed 50,000 IU of Vit D supplement (Zahravi Pharmaceutical Company, Tabriz, Iran) once a week, while AT and C consumed a placebo with the same taste, color, and shape (Barij Essence Pharmaceutical Company, Kashan, Iran) [19, 23]. Additionally, a self-reported three-day food record [analyzed by nutritionist IV software (First Databank, San Bruno, CA)] was used at the baseline in weeks 2, 4, 6, and 8 of the intervention to describe the daily diet precisely.

## Measurements

### Anthropometric and body composition

The initial measurements were taken 3 days before and after the intervention. Height and Waist Circumference (WC) was measured to the nearest 0.5 cm using a stadiometer (DETECTO, Model 3PHTROD-WM, USA) and a non-elastic tape measure. The Bioelectrical impedance analysis (BIA; Zeus 9.9 PLUS; Jawon Medical Co., Ltd., Kungsang Bukdo, South Korea) was used to measure Bodyweight (BW), Body Mass Index (BMI), Waist–Hip Ratio (WHR), and Body Fat Percentage (BFP) after at least 12-h of fasting between 8 and 9 in the morning. The participants were asked not to participate in intensive physical activities 72 h before the measurements.

### Blood sampling

Forty-eight hours before and after the intervention, 15 ml of fasting blood was obtained from the median cubital vein. Serum levels of lipid profile (Triglyceride (TG), Total Cholesterol (TC), High-Density Lipoprotein (HDL), and Low-Density Lipoprotein (LDL); using Hitachi kits, Tokyo, Japan). Additionally, liver enzymes (Alanine Aminotransferase (ALT), Aspartate Aminotransferase (AST), and Gamma-Glutamyl Transferase (GGT); with the ELISA method (Greiner Bio-One Kit, Germany)) were measured.

### Isolation of PBMCs

The Peripheral Blood Mononuclear Cells (PBMCs) isolated from blood samples were used to examine the gene expression levels associated with inflammation [24]. The gene expression of IL-6, IL-10, CD27, CXCL13, IFN-γ, and TGF-β1 were assessed as the primary outcomes due to their role in the signaling pathway of diabetic low-grade inflammation and secondary outcomes [25–27]. Blood samples were mixed through 3-part diluted blood to 2-part Ficoll-Hypaque and centrifuged for 30-min at 500×g for PBMCs isolation. Then, using a sterile Pasteur pipet, PBMCs were carefully aspirated, and 10 mL of phosphate-buffered saline (PBS) was added and centrifuged for 10 minutes at 400 g, followed by Hanks balanced salt solution and Percoll. The cloudy layer in the top 5 mm was transferred into a separate tube for further analysis after centrifugation for 25 minutes at 370 g, 25 °C.

### RNA extraction and real-time PCR

Total RNAs were extracted from one aliquot of 5 × 106 unactivated PBMCs with the trizol reagent (Invitrogen, USA) using an RNX-plus kit (Cinnacolon, Tehran, Iran) based on the manufacturer’s instructions. The RNA was quantified using a UV spectrophotometer, which revealed no contamination with protein or DNA (OD 260/280 ratio between 1.7 and 2.1), followed by reverse transcription to the cDNA library (via Moloney murine leukemia virus reverse transcriptase). Then, glyceraldehyde-3-phosphate dehydrogenase primers as housekeeping gene and quantitative RT-PCR method (LightCycler technology, Roche Diagnostics, Rotkreuz, Switzerland; SYBR green detection and Amplicon Kit), the gene expression of IL-6, IL-10, CD27, CXCL13, IFN-γ, and TGF-β1 were evaluated. Additionally, the Primer Express (Applied Biosystems, Foster City) and Beacon designer (Takaposizt, Tehran, Iran) software were used to design the primers and Pffafi or 2 − 11CT method to calculate relative transcription levels (Table 2).

**Table 2:** Forward and Reverse Primers Used for Real-Time Quantitative PCR

Gene	Primer	Product size (bp)	Annealing temperature (C)
IL-6	F: GGTACATCCTCGACGGCATCT R: GT GCCTCTTTGCTGCTTTCAC	250	60
IL-10	F: AAGGCAGTGGAGCAGGTGAA R: CCAGCAGACTCAATACACAC	250	58
CD27	F: TGTCGGCACTGTAACTCTGGTCT R: CCTGCACTGCCAGCCAT	250	59
CXCL13	F: GCTTGAGGTGTAGATGTGTCC R: CCCACGGGGCAAGATTTGAA	150	59
IFN-γ	F: GGCATTTTGAAGAATTGGAAAG R: TTTGGATGCTCTGGTCATCTT	286	60
TGF-β1	F: CCCAGCATCTGCAAAGCTC R: GTCAATGTACAGCTGCCGCA	286	60

### Statistical analysis

The SPSS software (IBM Corp., Armonk, NY, USA) was used to perform the statistical analyses. Mean ± standard deviation (SD) was used to present the data. The Shapiro–Wilk test was used to check the normal distribution of the variables. One-way analysis of variance (ANOVA) and Tukey’s post hoc were used to analyze the between-group differences. The paired sample t-test analyzed within-group differences at *P*-values less than 0.05.

## Results

Table 3 presents the Mean ± SD of baseline levels of anthropometric and physiologic variables and their between-group comparison. The results of the one-way ANOVA show no significant differences in the mean of WC, BW, BMI, BFP, WHR, FBG, and insulin in the pre-test between groups.

**Table 3:** Mean ± SD of anthropometric and physiologic variables before the intervention in patients with T2DM

Variables	AT + Vit D	AT	Vit D	C	*P*-Value
Age (years)	48.32 ± 2.23	47.13 ± 3.12	49.10 ± 1.23	48.27 ± 2.17	0.540
Height (cm)	159.14 ± 2.13	161.17 ± 2.21	160.21 ± 2.28	162.12 ± 3.18	0.304
WC (cm)	10.13 ± 3.23	9.10 ± 2.13	11.15 ± 1.89	10.25 ± 3.26	0.215
BW (Kg)	75.11 ± 2.12	76.12 ± 3.14	74.18 ± 3.09	75.09 ± 2.17	0.451
BMI (kg/m^2^)	29.66 ± 1.25	29.30 ± 1.43	28.90 ± 1.23	28.57 ± 1.27	0.356
BFP (%)	32.16 ± 1.56	31.11 ± 2.14	30.23 ± 1.56	29.76 ± 2.08	0.084
WHR (cm)	0.94 ± 0.01	0.93 ± 0.03	0.92 ± 0.04	0.92 ± 0.02	0.078
Insulin (μU/mL)	7.87 ± 0.29	7.59 ± 0.56	6.89 ± 0.23	6.73 ± 0.33	0.117
FBG (mg/dl)	169.76 ± 2.05	158.23 ± 2.41	150.18 ± 1.44	153.33 ± 2.54	0.063

Data analysis was done by the analysis of one-way analysis of variance and least significant difference post-hoc Tukey’s test after adjustment for baseline values

*AT* Aerobic Training, *Vit D* Vitamin D Supplementation, *C* Control, *WC* Waist Circumferences, *BW* Bodyweight, *BMI* Body Mass Index, *BFP* Body Fat Percentage, *WHR* Waist To Hip Ratio, *FBG* Fasting Blood Glucose

Table 4 shows the between-group comparisons of the lipid profile (TC, TG, LDL, and HDL), liver enzymes (AST, ALT, and GGT), and serum 25-OH-Vit D. The results of the t-test indicate significant differences in the post-test compared to the pre-test. After 8 weeks, TC, TG, LDL, AST, ALT, and GGT decreased significantly in AT+Vit D, AT, and Vit D but not significantly in C (Table 3). Based on the results, there was a significant increase in the HDL and 25-OH-Vit D after 8 weeks in AT+Vit D, AT, and Vit D groups. Comparing the pre-test to the post-test, no significant differences were found in HDL levels in the C group. The results of one-way ANOVA indicate significant between-group differences in the **Δ** (post-pre) of variables mentioned previously.

**Table 4:** Mean ± SD of lipid profile and liver enzymes before and after the 8-week intervention in patients with T2DM

Variables	AT + Vit D	AT	Vit D	C	*P*-Value
AST (U/L)					
Before	35.17 ± 2.29	34.59 ± 2.56	36.89 ± 2.23	35.73 ± 1.30	
After	25.13 ± 1.11	27.73 ± 1.24	30.20 ± 1.22	36.97 ± 2.23	
P^†^	0.001^*^	0.022^*^	0.039^*^	0.398	
Δ	−10.04 ± 1.18^μ€β^	−6.86 ± 1.32^€β^	−5.69 ± 1.01^β^	1.24 ± 0.93	0.012^¥^
ALT (U/L)					
Before	33.36 ± 1.65	32.53 ± 2.28	34.18 ± 2.64	35.43 ± 1.50	
After	24.04 ± 2.07	26.10 ± 1.37	30.12 ± 1.73	35.89 ± 1.34	
P^†^	0.011^*^	0.024^*^	0.043^*^	0.624	
Δ	−9.32 ± 0.42^μ€β^	−6.43 ± 0.91 ^€β^	−4.06 ± 0.91^β^	0.46 ± 0.16	0.002^¥^
GGT (U/L)					
Before	38.34 ± 1.29	36.97 ± 2.26	36.23 ± 1.19	37.35 ± 1.26	
After	26.24 ± 2.23	28.07 ± 1.17	30.14 ± 2.31	38.71 ± 2.15	
P†	0.001*	0.014*	0.029*	0.287	
Δ	−12.10 ± 0.94 ^μ€β^	−8.90 ± 1.09 ^€β^	−6.09 ± 1.12 ^β^	1.36 ± 0.89	0.003 ¥
TC (mg/dl)					
Before	187.19 ± 3.87	175.27 ± 5.11	179.04 ± 2.94	186.14 ± 3.26	
After	164.17 ± 1.21	162.14 ± 3.54	169.36 ± 4.13 ^β^	188.21 ± 1.49	
P^†^	0.001^*^	0.001^*^	0.012^*^	0.282	
Δ	−23.02 ± 2.66^μ€β^	−13.13 ± 1.57^β^	−9.68 ± 1.19^β^	2.07 ± 1.77	0.001 ^¥^
TG (mg/dl)					
Before	163.42 ± 5.07	160.15 ± 3.07	155.43 ± 4.64	153.08 ± 3.26	
After	146.13 ± 2.92^μ€β^	150.25 ± 2.23	147.32 ± 2.76^β^	156.35 ± 2.38	
P^†^	0.001^*^	0.003^*^	0.018^*^	0.216	
Δ	−17.29 ± 2.15^μ€β^	−9.90 ± 0.84^€β^	−8.11 ± 1.88^β^	3.27 ± 0.88	0.003 ^¥^
LDL (mg/dl)					
Before	143.12 ± 4.34	140.26 ± 3.56	147.12 ± 6.47	142.14 ± 3.23	
After	123.07 ± 2.33	128.27 ± 2.06	139.02 ± 2.02	144.17 ± 2.66	
P^†^	0.001^*^	0.001^*^	0.017^*^	0.294	
Δ	−20.05 ± 2.01^μ€β^	−11.99 ± 1.50^€β^	−8.10 ± 4.45^β^	2.03 ± 0.57	0.002 ^¥^
HDL (mg/dl)					
Before	31.14 ± 2.63	32.29 ± 1.38	33.18 ± 1.27	32.54 ± 1.29	
After	42.07 ± 1.82^μ€β^	38.06 ± 2.11^β^	37.92 ± 1.42^β^	30.36 ± 2.15	
P^†^	0.001^*^	0.031^*^	0.042^*^	0.092	
Δ	10.93 ± 0.81^μ€β^	5.77 ± 0.73^β^	4.74 ± 0.15^β^	−2.28 ± 0.86	0.026 ^¥^
serum 25-OH-Vit D (ng/mL)					
Before	22.22 ± 1.29	21.45 ± 2.57	23.17 ± 1.02	23.20 ± 3.41	
After	39.67 ± 0.84	28.26 ± 1.62	34.18 ± 1.24	22.70 ± 1.64	
P^†^	0.001^*^	0.002^*^	0.001^*^	0.111	
Δ	17.45 ± 0.45^μ€β^	6.81 ± 0.95^€β^	11.01 ± 0.22^β^	−0.50 ± 1.77	0.001 ^¥^

Data analysis was done by one-way analysis of variance and least significant difference post-hoc Tukey’s test after adjustment for baseline values

*AT* Aerobic Training, *Vit D* Vitamin D Supplementation, *C* Control Group, Δ pos-pre

P^†^Statistical analysis was done by paired sample t-test

^*^Significantly different in comparison pre and post within the groups

^¥^Significantly different comparing Δ between groups

^μ^Significantly different compared with AT

^€^Significantly different compared with Vit D

^β^Significantly different compared with C

Compared to the C group, AT+Vit D, AT, and Vit D significantly decreased TC, TG, LDL, AST, and ALT, GGT while increased HDL and 25-OH-Vit D. Additionally, the AT+Vit D group significantly decreased TC, TG, LDL, AST, ALT, and GGT levels and increased HDL, and 25-OH-Vit D levels compared to the individual AT and Vit D groups. The results also indicate decreased TC, TG, LDL, AST, and ALT and increased HDL, and 25-OH-Vit D in AT compared to Vit D, although they were not significant.

**Fig. 2:**
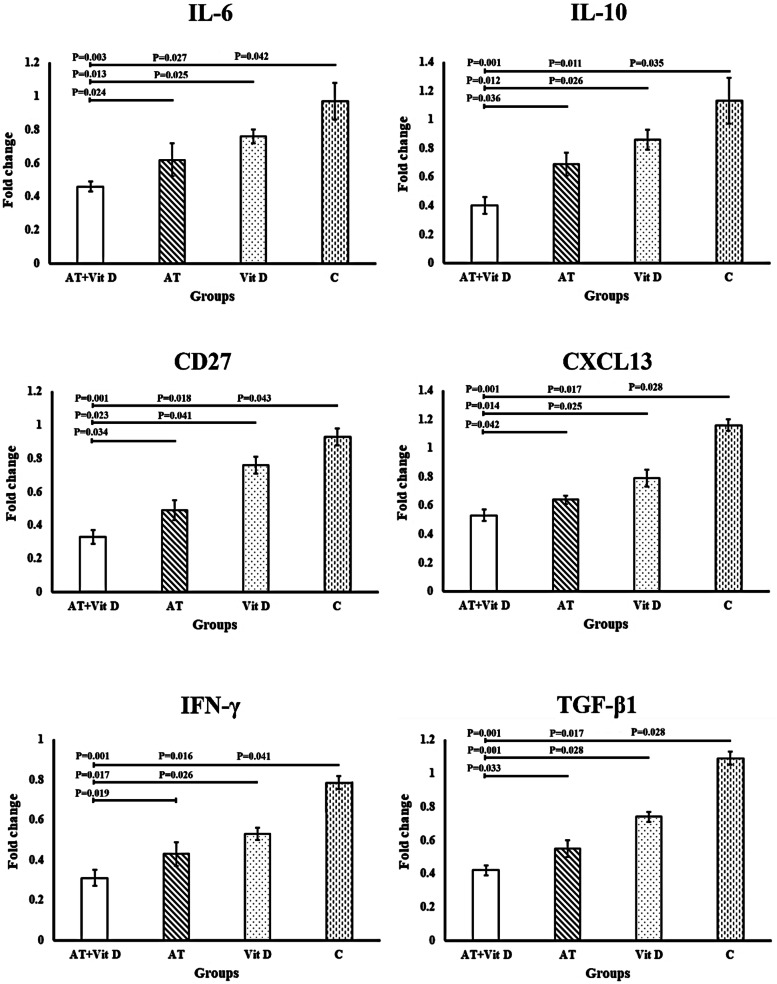
Effect of aerobic training and vitamin d supplementation on IL-6, IL-10, CD27, CXCL13, IFN-γ, and TGF-β1 in patients with T2DM. IL-6: Interleukin-6; IL-10: Interleukin-10; CD27: Cluster of Differentiation 27; CXCL13: Chemokine (C-X-C motif) Ligand 13; IFN-γ: Interferon-Gamma a; TGF-β1: Transforming Growth Factor-Beta 1. *P*-value was obtained from One-way ANOVA and Tukey’s post hoc test. Data are means ± standard deviation

Based on the results of the t-test, gene expressions of IL-6, IL-10, CD27, CXCL13, IFN-γ, and TGF-β1 were downregulated significantly after 8 weeks of AT+Vit D, AT, and Vit D while upregulated significantly in C group (Fig. 2). Furthermore, compared to C group AT+Vit D, AT, and Vit D significantly downregulated IL-6 (*P* = 0.003; *P* = 0.027; *P* = 0.042), IL-10 (*P* = 0.001; *P* = 0.011; *P* = 0.035), CD27 (*P* = 0.001; *P* = 0.018; *P* = 0.043), CXCL13 (*P* = 0.001; *P* = 0.0017; *P* = 0.028), IFN-γ (*P* = 0.001; *P* = 0.016; *P* = 0.041), and TGF-β1 (*P* = 0.001; *P* = 0.019; *P* = 0.037) gene expressions. Also, significant differences were observed in the gene expressions of IL-6 (*P* = 0.013; *P* = 0.025), IL-10 (*P* = 0.012; *P* = 0.026), CD27 (*P* = 0.023; *P* = 0.041), CXCL13 (*P* = 0.014; *P* = 0.025), IFN-γ (*P* = 0.017; *P* = 0.026), and TGF-β1 (*P* = 0.001; *P* = 0.028) in the AT+Vit D compared to individual AT or Vit D, respectively. Moreover, AT significantly downregulated the gene expressions of IL-6 (*P* = 0.024), IL-10 (*P* = 0.036), CD27 (*P* = 0.034), CXCL13 (*P* = 0.042), IFN-γ (*P* = 0.019), and TGF-β1 (*P* = 0.033) than Vit D (Fig. 2).

## Discussion

The present study investigated whether separate or combined AT and Vit D induce more significant changes in the liver enzymes, lipid profile, and inflammation-related gene expression in individuals with T2DM. We showed that 8-week AT and Vit D significantly improved lipid profile accompanied by a significant decrease in liver enzymes (ALT and AST). In line with our findings, previous research has shown that AT is an essential component of T2DM management (31) and is associated with improved hepatic risk factors (i.e., ALT, AST, and GGT) [19]. Interestingly, combined AT+ Vit D appears to produce more beneficial changes in lipid profile and liver enzymes than single interventions. Consistent with our findings, Vit D and AT have significantly reduced BW, BMI, visceral fat, liver enzymes, and improved lipid profile in the rat [28] and human [19] models. Improved liver enzymes following exercise might result from weight loss pathophysiological changes that lead to improved insulin sensitivity, reduced free fatty acid transfusion into the liver, and decreased inflammatory mechanisms [29, 30]. The improvement of lipid profile could be due to the use of fat as an energy substrate both in exercising and recovery states. Also, exercise appears to increase lipoprotein A, lipoprotein lipase (LPL) enzyme levels, and catabolism of the lipid as a consequence [31–33]. Additionally, Carmeliet et al. [34] reported that Vit D reduces the liver secretion of triglyceride via increasing intracellular calcium, fecal excretion of bile acids, and creating calcium-fatty acid soap [34, 35]. Hypovitaminosis D may generally interfere with the proper function of insulin receptors and GLUT-4 in target tissues, probably via increasing parathyroid hormone levels [36, 37]. Therefore, combined AT and Vit D might be beneficial for inducing liver enzyme and lipid profile improvements in T2DM patients through the mechanisms mentioned above.

This study showed downregulated IL-6, IL-10, CD27, CXCL13, IFN-γ, and TGF-β1 gene expression after 8 weeks in all three experimental groups compared to the control group. Although AT and Vit D groups induced significant anti-inflammatory effects in human-derived PBMCs in the present study, these alterations were more significant in the AT+ Vit D group. Thus, combined AT+ Vit D was significantly more beneficial in downregulating IL-6, IL-10, CD27, CXCL13, IFN-γ, and TGF-β1 gene expression than AT and Vit D alone.

There is mounting evidence that those who are physically active or become physically active have a reduction in biomarkers associated with chronic inflammation [38–40]. The primary mechanisms through which exercise training promotes anti-inflammatory effects are still unknown. However, some intriguing possibilities might include: reduced visceral adiposity [41, 42], increased heat shock proteins releasement [43, 44], decreased local hypoxia and ischemia [44], reduced adipocytes infiltration via macrophages [41], altered immune cell phenotype, and lower monocyte toll-like receptor 4 (TLR4) expression [38]. In the absence of regular exercise training, these reciprocal adjustments tend to produce a vicious cycle leading to increased fat mass and inflammation because exercise-induced fat loss and reduced adiposity mediate the expression of inflammatory genes.

Regardless of the contradictory results in the literature, Vit D is also believed to modulate innate immune response and inflammation [45–47]. Considering the existence of Vit D Receptor (VDR) in the pancreas [48], vascular endothelial cells and PBMC [49], skeletal muscle cells, and other tissues [50] modulating a wide range of physiological functions are expected. The downstream signaling cascades of VDR in PBMCs remain unclear; however, recent studies indicate the alteration of inflammation pathways following Vit D supplementation. The activated VDR then might act as a transcription factor [51, 52] or modulate other unidentified molecules inducing the post-translational modifications or inhibitory effects [53] that regulate target gene expression [47] and reduce the level of inflammatory factors, in turn.

Finally, it seems that AT and Vit D suppress target gene expressions through different mechanisms that might converge in the downstream elements, resulting synergically in reduced inflammation.

### Limitation of the study

This study was a placebo-controlled, single-blind, randomized trial with a low dropout rate evaluating the alterations of gene expression in human subjects. The small sample size is one of the limitations of this study thus concluding a conclusive response is premature. Therefore, we suggest investigating the same intervention in larger sample sizes and other tissues (e.g skeletal muscle cells). Also, it is suggested to investigate the level of inflammatory biomarkers in the diabetes population without Vit D deficiency or in animal models to better control Vit D intake as well as, exposure to UV.

## Conclusion

Finally, any event that could modulate the vicious cycle of inflammation could play a key role in improving low-grade systemic inflammation in T2DM patients. As a result, AT+ Vit D could drive the balance to favor inhibition of inflammation and break this vicious cycle by improving lipid profile and liver enzymes while downregulating gene expression of inflammation-related factors. As a result, AT+ Vit D could be considered an ideal therapy for managing T2DM.

## Data Availability

The datasets generated and analyzed during the current study are not publicly available due to ongoing data analysis but are available from the corresponding author on reasonable request.

## Abbreviations

AT: Aerobic Training
Vit D: Vitamin D
T2DM: Type 2 Diabetes Mellitus
FBG: Fasting Blood Glucose
IL-6: Interleukin-6
IL-10: Interleukin-10
CD27: Cluster of differentiation 27
CXCL13: Chemokine (C-X-C motif) Ligand 13
IFN-γ: Interferon-Gamma
TGF-β1: Transforming Growth Factor-Beta 1
